# The Association between Fear of Crime, Educational Attainment, and Health

**DOI:** 10.3390/epidemiologia4020016

**Published:** 2023-04-30

**Authors:** Gloria Macassa, Cormac McGrath, Katarina Wijk, Mamunur Rashid, Anne-Sofie Hiswåls, Joaquim Soares

**Affiliations:** 1 Department of Public Health and Sports Science, Faculty of Occupational and Health Sciences, University of Gävle, 80176 Gävle, Sweden; 2 EPI Unit—Instituto de Saúde Pública, Universidade do Porto, 4050-600 Porto, Portugal; 3 Laboratório para a Investigação Integrativa e Translacional em Saúde Populacional (ITR), 4050-600 Porto, Portugal; 4 Department of Education, Stockholm University, 10691 Stockholm, Sweden; 5 Centre for Research and Development, Uppsala University, 80187 Gävle, Sweden; 6 Department of Occupational Health Sciences and Psychology, University of Gävle, 80176 Gävle, Sweden; 7 Department of Public Health and Caring Sciences, Uppsala University, 75123 Uppsala, Sweden; 8 Department of Health Sciences, Mid-Sweden University, 85170 Sundsvall, Sweden; 9 Department of Psychology, Universidade Europeia, 1500-210 Lisbon, Portugal

**Keywords:** fear of crime, educational attainment, women, self-rated health, anxiety, Gävleborg

## Abstract

Fear of crime is an important public health problem that impacts people’s quality of life, health, and wellbeing, and causes mental health ailments (e.g., anxiety). This study aimed to determine whether there was an association between fear of crime, educational attainment, and self-rated health and anxiety among women residing in a county in east-central Sweden. A sample (*n* = 3002) of women aged 18–84 years surveyed in the Health on Equal Terms survey carried out in 2018 was included in the study. Bivariate and multivariate regression analysis was performed on the relationship between the composite variables fear of crime, educational attainment, and self-rated health and anxiety. Women with primary education or similar who reported fear of crime had increased odds of poor health (odds ratio (OR) 3.17; 95% confidence interval (CI) 2.40–4.18) compared with women with primary education/similar and no fear of crime (OR 2.90; CI 1.90–3.20). A statistically significant relationship persisted in the multivariate analysis after controlling for other covariates, although the odds were reduced (OR 1.70; CI 1.14–2.53 and 1.73; CI 1.21–2.48, respectively). Similarly, in the bivariate analysis, women who reported fear of crime and who only had primary education had statistically significant odds of anxiety (OR 2.12; CI 1.64–2.74); the significance was removed, and the odds were reduced (OR 1.30; CI 0.93–1.82) after adjusting for demographic, socioeconomic, and health-related covariates. Women with only primary education or similar who reported fear of crime had higher odds of poor health and anxiety compared with those with university education or similar, with and without fear of crime. Future studies (including longitudinal ones) are warranted—on the one hand, to understand possible mechanisms of the relationship between educational attainment and fear of crime and its consequences to health, and on the other, to explore low-educated women’s own perceptions regarding factors underlining their fear of crime (qualitative studies).

## 1. Introduction

Fear of crime (defined as “the fear of being a victim of crime” rather than actually being a victim of crime) [1] is an important public health and social problem across societies [2,3,4,5,6], which affects people’s quality of life, health, and wellbeing and causes mental health ailments (e.g., anxiety) [7,8]. It is considered an emotional and psychological reaction to an event [9], p. 25. There is agreement that fear of crime encompasses feelings, thoughts, and behaviors that need to be distinguished from those linked to actual experience of crime [2,10].

Research has found gender differences in fear of crime [11,12,13,14], with women reporting being more afraid of crime than men, no matter how, when, or where fear of crime is measured [13,14]. Four possible theoretical explanations have been put forward for why women are more likely to fear crime, even though women are less likely to be victimized compared with men [13,14]. These explanations include the perceived threat of rape, patriarchy, differential socialization, and physical vulnerability [13,14]. Moreover, several other factors are known to influence fear of crime, such as age, race, ethnicity, socio-economic status (e.g., education, income) [15,16,17,18], and victimization (direct as well as indirect) [19,20,21].

One of the socio-economic status-related factors affecting fear of crime we identified is educational attainment, which is central to this study. Educational attainment is defined as the “highest level of education that an individual has completed” [21], p. 6, and is considered to positively impact life-long success both in the short and long term in relation to preventing the likelihood of involvement in criminal behavior [21], p. 6. Lack of educational attainment can create barriers to aspirations for employment, creating conditions conducive to criminal behavior (especially for those who might never get employed) and vulnerability to exploitation by others and potential secondary criminal behavior [21], p. 6. Empirical evidence shows an association between educational attainment and fear of crime, indicating that individuals with low education are likely to be more fearful of crime compared with those with higher levels of education [22,23]. For instance, one study found that participants with no education or only primary school education were three times more fearful of crime than those with higher education, and those with secondary education were more than four times more likely to be fearful of crime than their higher-educated counterparts [24]. Franklin et al. found that individuals who had lower levels of education (and were poor) were more likely to report increased fear of crime [18].

Both fear of crime and educational attainment have consequences for health and wellbeing. Fear of crime has consequences for people’s behavioral health (e.g., potentially leading to substance use and alcohol consumption) [9,25], physical health (e.g., causing reduced mobility and physical activity, as well as potential fight and flight syndrome, which may cause physiological responses that are associated with chronic illness and poor self-reported health) [2,25,26], and psychological health (e.g., leading to anxiety and depression) [27,28,29]. On the other hand, educational attainment is also associated with health outcomes [30,31,32,33] and is considered an important determinant of health disparities [34,35], especially inequities in health, which are considered unjust and unfair [36].

As elsewhere, in Sweden, fear of crime has been found to be higher in women than in men nationally [37] as well as in some regions/counties [38,39]. Furthermore, an association between fear of crime and health outcomes has been observed [2]. However, few studies, if any, have simultaneously assessed the relationship between fear of crime and educational attainment, on the one hand, and fear of crime and health-related outcomes, on the other, in Sweden. Specifically, in Gävleborg County, which has low levels of education compared with the national average, this topic would be of interest. Therefore, to fill this knowledge gap, this study sought to study how fear of crime and educational attainment together impact health-related outcomes. The following research questions were addressed: (a) What is the relationship between fear of crime/educational attainment and self-rated health? (b) Is there an association between fear of crime/educational attainment and anxiety?

## 2. Materials and Methods

### 2.1. Study Setting

The study took place in Gävleborg County, which is situated in east-central Sweden and had an estimated population of 287,000 inhabitants in 2022 [40]. The county has ten municipalities and small towns, and its administrative center is the Municipality of Gävle, with 103,493 inhabitants (2022 population) [41]. As mentioned above, the county’s education level is lower than the national average [20]. For instance, at the county level, a 2016 report showed that, among those aged 20–64 years, 37% of women as compared with 24% of men had completed high school education. Furthermore, 13% of women had only completed primary education, compared with 17% of men [42].

### 2.2. Data Collection and Procedure

This study is based on secondary data for Gävleborg, gathered using the Health on Equal Terms (HET) national cross-sectional survey from 2018. The survey was carried out by the Swedish Agency for Public Health in collaboration with the county councils. More details about the survey procedures, as well as the questionnaire and technical reports, can be found elsewhere [2,43,44]. The questionnaire was distributed by Statistics Sweden in collaboration with Gävleborg County Council and the Swedish National Public Health Agency [43]. The 2018 HET survey was performed first in a national, non-stratified sample, followed by a regional sample stratified by age, gender, county, and municipality [43]. In Gävleborg county, the HET survey targeted a total of 12,000 persons aged 16–84. Of these, 5599 returned the questionnaire by post or completed it online (43% response rate) [43]. There were no inclusion or exclusion criteria, and item non-response was handled using complete case analysis. All HET questionnaires, including the one in the 2018 survey, covered an array of topics such as health, health care, psychosocial and material conditions, as well as health behaviors [43,44].

The survey data were collected by linking the respondents’ personal identity number to individual-level socio-demographic data such as annual income (for 2016) and education level retrieved from the total population registers of Statistics Sweden [43]. This study is based on the data on women (*n* = 3002), who were the majority of respondents.

### 2.3. Study Variables

The outcome variables in this study are self-rated health and anxiety. Self-rated health was dichotomized into bad (if respondents answered “fair”, “bad”, or “very bad”) and good (if respondents answered “good” or “very good”). Anxiety was classified as yes (worry and anxiety) and no (no worry or anxiety). The main explanatory variables in the study were fear of crime and educational attainment. Fear of crime was based on the question of whether respondents avoided going out alone for fear of being assaulted, robbed, or otherwise victimized. In the study, fear of crime was dichotomized into yes (“yes, sometimes” and “yes, often”) and no. Educational attainment data are based on information obtained from Statistics Sweden (valid for 2016) using the Swedish standard classification of education [45] into primary education/similar; secondary education/similar; and university education/similar. To address the main objective of the study, a composite variable of fear of crime and educational attainment was created consisting of the following levels: fear of crime/low education; no fear of crime/low education; fear of crime/intermediate education; no fear of crime/intermediate education; fear of crime/high education; and no fear of crime/high education.

Covariates included age (18–29, 30–44, 45–64, and 65–84 years); country of birth (Sweden, and other); work status (employed, student/intern, unemployed, and retired); civil status (unmarried and not cohabitating, married or cohabitating, divorced, and widowed); social support from friends and family (“yes” or “no”); practical support (e.g., getting advice, help with food shopping and repairs) (“yes” or “no”); economic strain (“yes” (for those answering yes: “yes, always”, or “yes, more than once”) and “no”); risk consumption of alcohol (“yes” or “no”); long-standing illness (“yes” and “no”); and experience of any type of violence, physical or psychological (“yes” or “no”); general trust (e.g., trust in institutions). The variable “any type of violence” was created because small numbers reported both physical and psychological violence.

### 2.4. Statistical Analyses

First, descriptive statistics of the sample were carried out (see Table 1). Then, a logistic regression was carried out to test bivariate (Model 1) and multivariate (Model 2) relationships between the composite variables fear of crime and educational attainment, and self-rated health and anxiety. Model 1 analyzed only the relationships between the composite variable fear of crime and educational attainment and the two outcomes (self-reported health and anxiety). Then, in Model 2, an array of covariates (e.g., demographic, socio-economic, and health-related) were added to the previous relationships analyzed in Model 1. The composite variable fear of crime and educational attainment was weighted prior to the analysis. There was no collinearity among variables included in the regression analysis.

Results are presented as odds ratios (ORs) with 95% confidence intervals (CIs). All analyses were carried out using SPSS version 27 [46].

### 2.5. Ethical Considerations

The protocol of the study was approved by the Swedish Ethical Review Authority (Dnr: 2021-01195). Furthermore, before data collection, written informed consent was provided by all respondents. The study was conducted in accordance with the Helsinki Declaration.

## 3. Results

### 3.1. Background Characteristics of the Participants

The characteristics of the sample are described in Table 1. The overall prevalence of fear of crime in the studied sample (*n* = 3002) was 60.3% (women who reported fear of going out alone to avoid being assaulted, robbed, or otherwise molested) (see Table 1). Furthermore, the prevalence of fear of crime by educational attainment in the sample was as follows: fear of crime and primary education/similar 18.5% (*n* = 555); no fear of crime and primary education/similar 26.6% (*n* = 799); fear of crime and secondary education/similar 12.3% (*n* = 370); no fear of crime and secondary education/similar 17.3% (*n* = 520); fear of crime and university education/similar 11.1% (*n* = 332); and no fear of crime and university education/similar 14.2% (*n* = 426) (see Table 1).

### 3.2. Fear of Crime–Educational Attainment, and Self-Rated Health

Results of the regression analysis found a statistically significant association between fear of crime, –educational attainment and self-rated health. In the bivariate model, women with fear of crime and primary education/similar had an OR for poor health of 3.17 (95% CI 2.40–4.18), compared with women with no fear of crime and primary education/similar (OR 2.90; CI 1.90–3.20); with fear of crime and secondary education/similar (OR 1.80; CI 1.37–2.45); and with no fear of crime and secondary education/similar (OR 1.44; CI 1.08–1.92) (see Table 2, Model 1). However, controlling for other covariates reduced the OR to 1.70 (CI 1.14–2.53) for those with fear of crime and primary education/similar and to 1.73 (CI 1.21–2.48) for those with no fear of crime and primary education/similar, although the relationship continued to be statistically significant (see Table 2, Model 2).

In the same model, there was a statistically significant association between age, work position, income, and self-rated health. Women in the age group 30–44 had an OR for poor health of 2.49 (95% CI 1.36–4.56); corresponding figures were OR 3.18 (CI 1.70–5.98) for ages 45–64 and OR 2.40 (CI 1.17–1.54) for the age group 65–84 (see Table 2, Model 2). Moreover, compared with their employed counterparts, unemployed women, and women in the lowest income quartile (in SEK) had an OR for poor health of 2.36 (CI 1.24–4.48) and 2.73 (CI 1.84–4.05), respectively. Furthermore, women with no social support, no practical support, risky alcohol behavior, and long-standing illness had statistically significant odds of poor health (see Table 2, Model 2).

### 3.3. Fear of Crime, Educational Attainment, and Anxiety

Fear of crime and educational attainment were statistically related to anxiety. The results of the bivariate regression analysis showed that women who reported fear of crime and had only primary education/similar had an OR for anxiety of 2.12 (95% CI 1.64–2.74). Those with fear of crime and a university education/similar had an OR of 1.86 (CI 1.35–2.57) (see Table 3, Model 1). However, controlling for other variables in the multivariate regression analysis not only reduced the OR from 2.12 (CI 1.64–2.74) to 1.30 (CI 0.93–1.82) but also removed the statistical significance of the observed relationship between fear of crime and primary education/similar (see Table 3, Model 2).

Also in Model 2, the OR for anxiety among women who reported fear of crime and had a university education/similar decreased from 1.86 (95% CI 1.35–2.57) to 1.69 (CI 1.17–2.43) but remained statistically significant. Moreover, in the same model, women in the age groups 45–64 and 65–84 years had increased odds of anxiety. Additionally, women born outside Sweden, those with no social and practical support, and those with long-standing illness and/or risky alcohol behavior had higher odds of anxiety.

## 4. Discussion

To our knowledge, this is the first study to explore the relationship between fear of crime, educational attainment, and health outcomes in central Sweden. The prevalence of fear of crime in the analyzed sample was 60.3%, which is much higher than the 20.8% found among women in northern Sweden [38].

Results also indicated that fear of crime and educational attainment were statistically and significantly associated with poor self-rated health as well as anxiety. Women with only primary education/similar, with or without fear of crime, had increased odds of poor self-rated health. Moreover, the statistical significance did not decrease after adjusting for demographic, socio-economic, and health-related variables. Fear of crime and a university education/similar were likewise statistically related to anxiety, and the significance did not decrease after controlling for potential confounders. Similar results regarding the association between fear of crime and self-rated health and mental health (e.g., anxiety) have been found in both cross-sectional [27,47,48,49,50,51,52] and longitudinal studies [53]. Although very few studies have addressed the relationship between fear of crime, educational attainment, and health as in this study, there are indications from other studies across a variety of contexts that people with only primary or no education are three times as likely to be fearful of crime as those with higher education. Furthermore, as previously mentioned, those with only secondary education are more than four times as likely to be fearful of crime as their more educated counterparts [24,54,55,56,57].

In this study, the greatest OR for poor self-rated health and anxiety was observed among women with primary education/similar with fear of crime as compared with those with no fear of crime and a university education/similar. It is likely that these women reported poorer health and anxiety as a result of potential physical and emotional symptoms associated with their worry, irritability, or sleep difficulties [2,27,28]. It is argued that anxiety is likely to cause distress that, in turn, can interfere with the ability to fully enjoy life [2,27]. Furthermore, it is likely that due to their level of education, they lived in more disadvantaged neighborhoods, thus making it more difficult to feel safe going out alone. As already mentioned above, in this study, fear of crime was related to whether respondents avoided going out alone for fear of being assaulted, robbed, or otherwise victimized.

It has been argued that higher educational attainment not only promotes better health but also means that individuals are more likely to make better decisions for themselves and their families [58,59]. Moreover, educational attainment helps to improve employment opportunities as well as income; educated individuals are less likely to experience unemployment and have more perceived personal control, better health behaviors and better social standing and social support [60,61,62,63,64].

Elsewhere, Keane used education and income as proxies for socio-economic status and reported that women with lower socio-economic status were likely to be too worried to walk alone outside their home after dark or to be outside their home at night [65]. It has also been suggested that women with the lowest incomes (who are also likely to have low educational attainment) usually live in poverty, which is known to be linked to physical correlates (e.g., poor living and working conditions) that might expose them to an increased risk of victimization [66]. The argument here is that, because of poor financial resources, low-educated (and, hence, poor) women are likely to live and work in conditions that put them at constant risk (e.g., taking public transportation more often and after dark, as well as having jobs that can be less safe than the jobs of their more educated counterparts) [66].

However, some studies have found that people with high socio-economic status experienced more fear of crime, and the explanation was that they had more to lose [67]. Interestingly, the results of this study found that women who reported fear of crime and had a university education/similar had statistically significant and increased odds of anxiety even after controlling for other variables. Studies on the relationship between education inequality and fear of crime have reported a positive impact on feelings of unsafety and on risk perception [68,69].

It is difficult to compare the results of this study with those of other studies carried out in Sweden because, to our knowledge, none of them directly studied the dual impact of fear of crime and educational attainment on health-related outcomes. Nevertheless, there is evidence of an association between fear of crime and income (which is strictly related to education). For instance, three different studies found an association between income and fear of crime at individual and contextual levels [38,39,70]. Furthermore, in a study of individual and spatial dimensions of women’s fear of crime in Stockholm, Yates and Ceccato reported that women who experienced the most fear were young, single, with children, had been born abroad, and had been victimized [71]. The same study found that, for the most fearful women, the fear experienced in the neighborhood did not lead to place avoidance or acts of functional fear [71]. In addition, a comparative study concerning fear of crime among poor people in Sweden and Britain reported that the poor were more afraid than other socio-economic groups in both countries. However, in Britain, this was a result of the vulnerability of poor people on the labor market, while in Sweden, no explanation was given for why the poor were more fearful of crime [15].

Overall, the findings of this study concerning the relation between fear of crime, educational attainment, and health need further exploration, especially using qualitative studies, to better understand what factors are perceived as important by lower-educated women, particularly in the Gävleborg region, which has persistently had lower education rates across generations [72]. For instance, qualitative findings from elsewhere have suggested that local-level social determinants of fear of crime seem to be more important than those related to the physical environment [73,74]. In addition, longitudinal studies are needed to help uncover potential mechanisms linking fear of crime to educational attainment and physical and psychological health and wellbeing.

Moreover, our findings suggest that education needs to be part of potential interventions aimed at curbing fear of crime among women in the county. For example, it has been argued that interventions that can impact the broader social determinants of fear of crime (of which education is an important part) are more likely to reduce fear than those designed to prevent crime [73,74]. The former category of intervention can consequently contribute to improving health and wellbeing across different population groups, and specifically among those with low educational attainment.

### Methodological Considerations

The main strength of this study is that it used a large secondary dataset, which is likely to have decreased reporting bias. Furthermore, the study centered on women’s fear of crime, as women are known to report more fear of crime than their male counterparts [13,14]. Additionally, this study is one of the few that has explored the role of educational attainment in women’s fear of crime and its potential impact on health-related outcomes. Notwithstanding, the study has weaknesses. The study had a response rate of 43%, reflecting the general decline in response rates [75] in population-based surveys across Sweden [76] and globally [77]. However, for the 2018 HET surveys (as for all HET surveys), Statistics Sweden used weighting for estimating prevalence at the population level. For the 2018 HET survey, specifically in Gävleborg county, Statistics Sweden performed weighting using information from registers of the county’s total population [78]. Additionally, register data were used to deal with non-response bias across different groups of individuals in the county sample [44,79].

Furthermore, we analyzed secondary data that were not specifically collected for this study, meaning that some variables were not available for inclusion in the performed analyses. Moreover, we used cross-sectional data (simultaneous measurement of exposure and outcome), which approach does not allow for inferring causal relationships as temporal sequence cannot be established [80]. In addition, we did not examine fear of crime from a general perspective; we only looked at being afraid of going out alone (for fear of being assaulted, robbed, or otherwise victimized). Nevertheless, it has been argued that the type of fear measured in this study is related to social exclusion because being afraid to go out can limit a person’s freedom of movement [15]. Furthermore, we were unable to include neighborhood characteristics of the respondents’ place of residence (e.g., neighborhood design, social context, and safety aspects), which are known to mediate the relationship between fear of crime and health outcomes [81,82,83].

## 5. Conclusions

This study observed a statistically significant relationship between a composite exposure—fear of crime and educational attainment—and poor self-rated health and anxiety among women. Moreover, the study found that women who reported fear of crime and only had primary education/similar had much higher odds of poor self-rated health and anxiety, compared with those who did not report fear of crime and had a university education/similar. The statistically significant relationship remained for poor self-rated health but disappeared for anxiety after controlling for other covariates. The results warrant further research (of a longitudinal nature) to better understand possible mechanisms through which educational attainment affects fear of crime, as well as the consequences for health and wellbeing. In addition, qualitative studies are needed to identify what factors are perceived as important regarding fear of crime by the low-educated women themselves. This could be of importance for policymakers involved in crime prevention strategies at the county level.

## Figures and Tables

**Table 1:** Distribution of the variables in the sample of women using Health on Equal Terms (HET), 2018 survey data for Gävleborg County.

Variable	Number (*n* = 3002)	Percentage (%)
Fear of crime		
Yes	1810	60.3
No	1157	38.5
Missing	35	1.2
Composite variable: fear of crime and educational attainment		
Fear of crime and primary education/similar	555	18.5
No fear of crime and primary education/similar	799	26.6
Fear of crime and secondary education/similar	370	12.3
No fear of crime and secondary education/similar	520	17.3
Fear of crime and university education/similar	332	11.1
No fear of crime and university education/similar	426	14.2
Age group, years		
18–29	219	7.3
30–44	419	14.0
45–64	923	30.7
65–84	1318	43.9
Missing	123	4.1
Country of birth		
Sweden	2744	91.4
Other	258	8.6
Civil status		
Married	1358	45.2
Single	987	32.9
Divorced	431	14.4
Widowed	226	7.5
Work status		
Employed	1269	42.3
Student/intern	211	7.0
Retired	1088	36.2
Unemployed	72	2.4
Missing	362	12.1
Individual disposable income		
Q1 < 144	887	29.5
Q2 145–214	755	25.1
Q3 215–294	788	26.1
Q4 ≥ 295	552	18.4
Missing	20	0.7
Social support		
Yes	2698	89.9
No	277	9.2
Missing	27	0.9
Practical support		
Yes	2280	75.9
No	679	22.6
Missing	43	1.5
General trust		
No	679	22.6
Yes	2280	75.9
Missing	43	1.5
Long-standing illness		
No	1583	52.7
Yes	1342	44.7
Missing	77	2.6
Risky alcohol behavior		
No	2855	95.1
Yes	147	4.9
Violence (any type)		
No	2787	92.8
Yes	170	5.7
Missing	45	1.5

Q1, Q2, Q3, Q4 = first, second, third, and fourth quartiles.

**Table 2:** Odds ratios (ORs) with 95% confidence intervals (CIs) for the relationship between the composite variable fear of crime and educational attainment and self-rated health among women, based on Health on Equal Terms (HET) survey data for Gävleborg, 2018.

Variable	Model 1	Model 2
Fear of crime and educational attainment	OR CI (95%)	OR CI (95%)
Fear of crime and primary education/similar	3.17 (2.40–4.18) ***	1.70 (1.14–2.53) ***
No fear of crime and primary education/similar	2.90 (1.90–3.20) ***	1.73 (1.21–2.48) ***
Fear of crime and secondary education/similar	1.80 (1.37–2.45) ***	1.41 (0.94–2.11)
No fear of crime and secondary education/similar	1.44 (1.08–1.92) ***	1.32 (0.92–1.91)
Fear of crime and university education/similar	1.29 (0.90–1.86)	1.19 (0.75–1.88)
No fear of crime and university education/similar	1	1
Age group, years		
18–29		1
30–44		2.49 (1.36–4.56) ***
45–64		3.18 (1.70–5.98) ***
65–84		2.40 (1.17–1.54) ***
Country of birth		
Sweden		1
Other		1.02 (0.67–1.54)
Civil status		
Married		1
Single		1.20 (0.91–1.58)
Divorced		1.24 (0.91–1.69)
Widowed		0.96 (0.62–1.50)
Work status		
Employed		1
Student/intern		0.75 (0.44–1.31)
Retired		1.52 (0.99–2.33)
Unemployed		2.36 (1.24–4.48) ***
Individual disposable income		
Q1 < 144		2.73 (1.84–4.05) ***
Q2 145–214		1.82 (1.28–2.54) ***
Q3 215–294		1.46 (1.06–2.02) ***
Q4 ≥ 295		1
Social support		
Yes		1
No		1.76 (1.22–2.53) ***
Practical support		
Yes		1
No		2.22 (1.21–4.06) ***
General trust		
No		0.52 (0.40–0.68)
Yes		1
Long-standing illness		
No		1
Yes		6.40 (5.18–7.91) ***
Risky alcohol behavior		
No		1
Yes		1.71 (1.10–2.68) ***
Violence (any type)		
No		1
Yes		0.89 (0.57–1.40)

*** *p*-value < 0.001. Q1, Q2, Q3, Q4 = first, second, third, and fourth quartiles.

**Table 3:** Odds ratios (ORs) with 95% confidence intervals (CIs) for the relationship between the composite variable fear of crime, educational attainment, and anxiety among women in Gävleborg, Sweden, using data from the Health on Equal Terms (HET) survey, 2018.

Variable	Model 1	Model 2
Fear of crime and educational attainment	OR CI (95%)	OR CI (95%)
Fear of crime and primary education/similar	2.12 (1.64–2.74) ***	1.30 (0.93–1.82)
No fear of crime and primary education/similar	1.10 (0.86–1.40)	0.94 (0.69–1.28)
Fear of crime and secondary education/similar	1.99 (1.50–2.63) ***	1.36 (0.97–1.90)
No fear of crime and secondary education/similar	1.19 (0.92–1.54)	0.91 (0.67–1.23)
Fear of crime and university education/similar	1.86 (1.35–2.57) ***	1.69 (1.17–2.43) ***
No fear of crime and university education/similar	1	1
Age group, years		
18–29		1
30–44		0.84 (0.52–1.37)
45–64		0.53 (0.32–0.88) ***
65–84		0.34 (0.19–0.62) ***
Country of birth		
Sweden		1
Other		0.50 (0.35–0.74) ***
Civil status		
Married		1
Single		1.04 (0.82–1.31)
Divorced		1.06 (0.81–1.40)
Widowed		1.02 (0.52–1.30)
Work status		
Employed		1
Student/intern		1.08 (0.69–1.69)
Retired		1.33 (0.91–1.69)
Unemployed		1.36 (0.77–2.45)
Individual disposable income		
Q1 < 144		1.24 (0.88–1.75)
Q2 145–214		1.18 (0.88–1.60)
Q3 215–294		1.10 (0.85–1.43)
Q4 ≥ 295		1
Social support		
Yes		
No		1
Practical support		1.44 (1.03–2.00) ***
Yes		1
No		2.14 (1.03–2.00) ***
General trust		
No		0.53 (0.42–0.67) ***
Yes		1
Long-standing illness		
No		1
Yes		1.60 (1.33–1.92) ***
Risky alcohol behavior		
No		1.77 (1.18–2.67) ***
Yes		
Violence (any type)		
No		1
Yes		1.02 (0.68–1.52)

*** *p*-value < 0.001. Q1, Q2, Q3, Q4 = first, second, third, and fourth quartiles.

## Data Availability

The data are not publicly available owing to restrictions in the ethical approval for this study. Questions related to the data in this study should be directed to the corresponding author.
